# Delirium and Its Associations with Critical Care Utilizations and Outcomes at the Time of Hospital Discharge in Patients with Acute Brain Injury

**DOI:** 10.3390/medicina60020304

**Published:** 2024-02-10

**Authors:** Alex P. Raquer, Christine T. Fong, Andrew M. Walters, Michael J. Souter, Abhijit V. Lele

**Affiliations:** 1 Elson S. Floyd College of Medicine, Washington State University, Spokane, WA 99202, USA; alex.raquer@wsu.edu; 2 Department of Anesthesiology and Pain Medicine, University of Washington, Seattle, WA 98104, USA; cfong@uw.edu (C.T.F.); walters3@uw.edu (A.M.W.); msouter@uw.edu (M.J.S.); 3 Neurocritical Care Service, Harborview Medical Center, Seattle, WA 98104, USA

**Keywords:** delirium, acute brain injury, neurocritical care, outcome, pain, agitation, distress

## Abstract

*Background and Objectives*: We analyzed delirium testing, delirium prevalence, critical care associations outcomes at the time of hospital discharge in patients with acute brain injury (ABI) due to acute ischemic stroke (AIS), non-traumatic subarachnoid hemorrhage (SAH), non-traumatic intraparenchymal hemorrhage (IPH), and traumatic brain injury (TBI) admitted to an intensive care unit. *Materials and Methods*: We examined the frequency of assessment for delirium using the Confusion Assessment Method for the intensive care unit. We assessed delirium testing frequency, associated factors, positive test outcomes, and their correlations with clinical care, including nonpharmacological interventions and pain, agitation, and distress management. *Results*: Amongst 11,322 patients with ABI, delirium was tested in 8220 (726%). Compared to patients 18–44 years of age, patients 65–79 years (aOR 0.79 [0.69, 0.90]), and those 80 years and older (aOR 0.58 [0.50, 0.68]) were less likely to undergo delirium testing. Compared to English-speaking patients, non-English-speaking patients (aOR 0.73 [0.64, 0.84]) were less likely to undergo delirium testing. Amongst 8220, 2217 (27.2%) tested positive for delirium. For every day in the ICU, the odds of testing positive for delirium increased by 1.11 [0.10, 0.12]. Delirium was highest in those 80 years and older (aOR 3.18 [2.59, 3.90]). Delirium was associated with critical care resource utilization and with significant odds of mortality (aOR 7.26 [6.07, 8.70] at the time of hospital discharge. *Conclusions*: In conclusion, we find that seven out of ten patients in the neurocritical care unit are tested for delirium, and approximately two out of every five patients test positive for delirium. We demonstrate disparities in delirium testing by age and preferred language, identified high-risk subgroups, and the association between delirium, critical care resource use, complications, discharge GCS, and disposition. Prioritizing equitable testing and diagnosis, especially for elderly and non-English-speaking patients, is crucial for delivering quality care to this vulnerable group.

## 1. Introduction

Neurocritical care admissions encompass a diverse population of patients with various neurological conditions [1,2]. The epidemiology of neurocritical care admissions has evolved over the years, with an increasing number of individuals suffering from acute brain injuries (ABI), such as acute ischemic stroke (AIS), non-traumatic intraparenchymal hemorrhage (IPH), non-traumatic subarachnoid hemorrhage (SAH), and traumatic brain injury (TBI). Concurrently, delirium, an acute neuropsychiatric syndrome characterized by disturbances in consciousness and cognitive function, has gained prominence as a pivotal complication in these patients [3]. Understanding the prevalence and impact of delirium within this subset of neurocritical care admissions is essential for optimizing patient care and outcomes.

The American Psychiatric Association’s Diagnostic and Statistical Manual of Mental Disorders, fifth edition (DSM-5) lists five key features that characterize delirium: disturbance in attention (reduced ability to direct, focus, sustain, and shift attention) and awareness [4] as a result of cognitive deficits, attentional deficits, circadian rhythm dysregulation, emotional dysregulation, and alteration in psychomotor functioning [5]. The pathophysiology of delirium has been debated but likely involves a substrate (patients with a possible gene predisposition; apolipoprotein E), a milieu (environment, such as the intensive care unit), and factors such as location of neuronal injury [6,7], exposure to certain sedatives such as lorazepam [8,9,10], inflammation [11,12,13], and sepsis [14,15,16]. Delirium diagnosis and management have been subjects of substantial research, but the focus is primarily on broader populations within critical care settings [17] and in the perioperative setting [18]. Our knowledge is evolving in the context of ABI, yet many gaps persist. In a meta-analysis by Patel and colleagues of delirium in neurocritically ill patients, delirium was noted to be associated with increased hospital and intensive care unit length of stay and worse functional independence but not survival [19]. Delirium testing, prevalence, and associations specific to acute brain injuries, including AIS, IPH, SAH, and TBI, warrant further exploration. While we recognize that delirium is not exclusive to these conditions, their unique characteristics, etiologies, and clinical trajectories necessitate a dedicated investigation into the prevalence and factors associated with delirium in this population. To date, our understanding of the nuances of delirium in acute brain injury patients remains incomplete.

The existing literature on delirium in ABI has provided valuable insights, but critical gaps in our knowledge persist. These gaps include a limited understanding of who receives delirium testing, the prevalence of delirium within this population, and the complex interplay of factors that influence testing and outcomes. The need for further examination is evident in the quest to unravel the intricacies of delirium in acute brain injury patients. Moreover, elucidating these gaps holds promise for improving patient care and enhancing clinical outcomes in this vulnerable population. Considering these gaps and the pressing need for a comprehensive investigation, our study set forth specific aims to address critical questions in delirium among patients with ABI. We sought to elucidate the following: (a) Who among acute brain injury patients received delirium testing?; (b) What proportion of these patients tested positive for delirium?; (c) What factors influenced the likelihood of receiving delirium testing and the likelihood of testing positive?; (d) What are the associations between delirium and critical care utilization?; and (e) What is the association between delirium and discharge outcomes (disposition) in this population? Through these aims, we hope to contribute essential knowledge to inform clinical practice, improve patient outcomes, and, ultimately, enhance the care of individuals with ABI.

## 2. Materials and Methods

### 2.1. Institutional Review Board Review and Approval

This study (STUDY00009382) was reviewed by the Institutional Review Board of the University of Washington and approved on 2 July 2023. A waiver of consent was granted due to the retrospective study design.

### 2.2. Study Design, Patient Selection, Clinical Setting, and Data Collection and Curation

Our study included adult patients aged 18 years and over who were admitted with a diagnosis of AIS, IPH, SAH, or TBI between 1 January 2014 and 31 December 2022. Due to their clinical significance and distinct characteristics, we focused on these four ABI subtypes. Patients who were declared dead by neurologic criteria were excluded from the study to ensure the cohort represented individuals with a potential for clinical assessment and outcomes related to delirium. This study was conducted in a clinical setting encompassing a large academic medical center, a Level I trauma center, and a comprehensive stroke center. Notably, no specific institutional protocols were in place for determining the frequency of delirium testing or ordering nonpharmacological measures to reduce delirium risk. The bedside nurses used the Confusion Assessment Method (CAM)/CAM-ICU [20] to assess for delirium. These decisions were left to the discretion of the attending neurointensivist, who based them on individual patient assessments and clinical judgment—the absence of specific protocols allowed for a real-world exploration of clinical practices. Data collection involved a comprehensive review of medical records, including clinical notes, diagnostic reports, patient demographics, and billing codes. Delirium assessments, diagnostic criteria, and relevant clinical variables were extracted and curated for analysis. Strict adherence to patient confidentiality and data protection protocols was maintained throughout the data collection.

### 2.3. Exposure, Outcomes, and Covariates 

Our primary exposure variable was the diagnosis of one of the specified ABI subtypes (AIS, IPH, SAH, TBI). The primary outcomes of interest included the following: (1) The diagnosis and prevalence of delirium in patients with ABI. (2) The associations between delirium and clinical care utilization, including mechanical ventilation, intracranial pressure monitoring, and ICU length of stay. (3) The associations between delirium and discharge outcomes, such as discharge disposition. We also examined several covariates to assess their potential associations with delirium and outcomes. These covariates included age, sex, race/ethnicity, preferred language, and pre-admission co-morbidities, including psychoses, depression, alcohol abuse, drug abuse, and dementia. Our statistical analyses considered these covariates to understand better the factors influencing delirium and their impact on clinical care and discharge outcomes.

### 2.4. Statistical Analysis 

We initially conducted descriptive statistics, presenting counts and percentages to characterize the study population and critical variables. Subsequently, we performed univariable analysis to explore the relationships between individual covariates and delirium testing. The covariates selected included include pre-admission co-morbidities such as dementia [4], psychosis, alcohol and substance use, depression, admission Glasgow Coma Scale score (GCS), race/ethnicity (White/non-White), preferred language (English/non-English), intracranial pressure (ICP) monitoring, mechanical ventilation status, and exposure to benzodiazepines [4]. We categorized admission GCS, lowest GCS, and discharge GCS into three categories: GCS 13–15, GCS 9–12, and GCS 3–8.

Variables with *p* < 0.05 on univariable analysis were selected for multivariable analysis, and those with *p* < 0.05 and higher were dropped. We calculated adjusted odds ratios (aOR) and 95% confidence intervals for multivariable analysis to assess the associations between covariates, delirium testing, and delirium diagnosis while accounting for potential confounding factors. We assessed the goodness of fit of our models using the Hosmer–Lemeshow test. To control for multiple comparisons, we performed Bonferroni correction. A *p*-value of <0.05 was considered statistically significant. To ensure the statistical robustness of our analysis, we conducted a sample size estimation and power analysis for our study. Given the complexity of our multivariable logistic regression model with multiple covariates, we aimed to achieve a minimum of 10–20 events (delirium cases) per predictor variable to ensure reliable results. With ten covariates in our model and an assumed delirium prevalence of 43% [19], we estimated a minimum sample size of approximately 369 patients to meet this criterion. This sample size allowed us to comprehensively analyze the factors associated with delirium in patients with acute brain injuries while maintaining statistical power. We aimed for a power of at least 80% at a significance level (alpha) of 0.05 to detect meaningful associations in our logistic regression analyses. All statistical analyses were conducted using RStudio (Version: 2023.12.1+402) [21].

## 3. Results

### 3.1. Study Sample Creation

**Figure 1:**
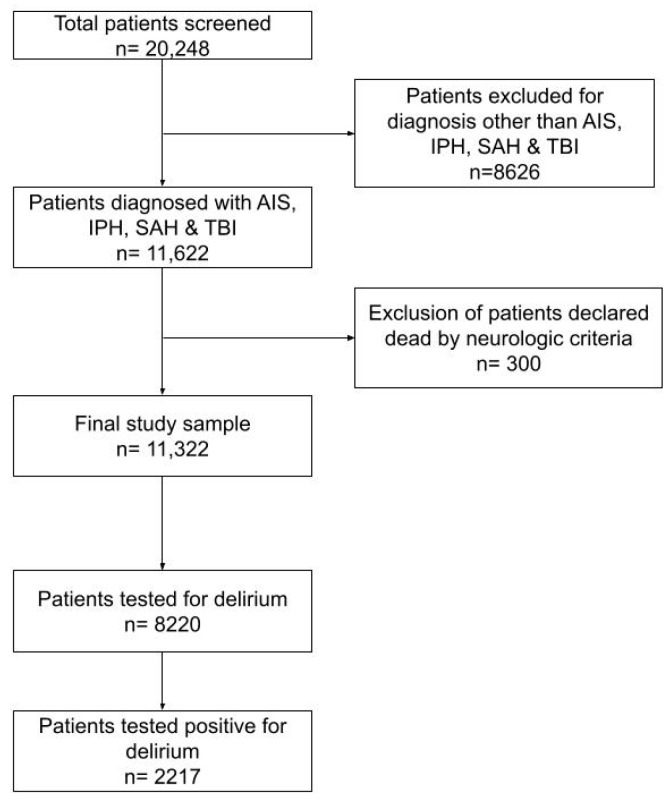
Flowchart with study sample selection. Abbreviations: AIS—acute ischemic stroke; IPH—spontaneous intraparenchymal hemorrhage; SAH—spontaneous subarachnoid hemorrhage; TBI—traumatic brain injury.

The study sample selection process is presented in Figure 1.

### 3.2. Study Sample Characteristics 

The cohort characteristics are presented in Table 1. 

### 3.3. Delirium Diagnostic Test Performance

**Figure 2:**
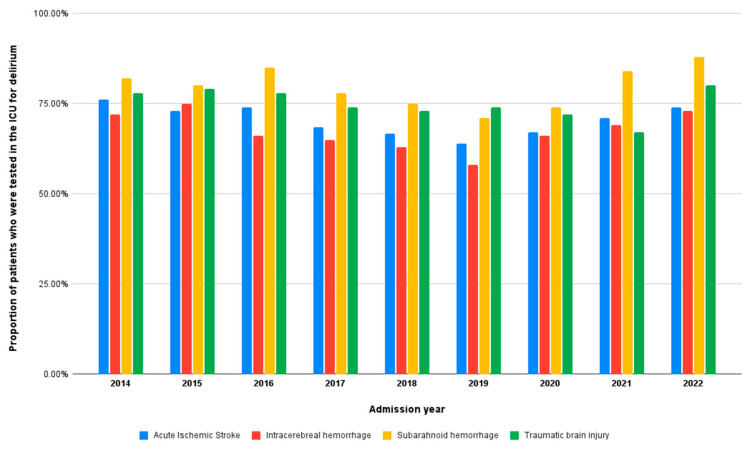
Delirium testing frequency among acute brain injury patients over the study period.

In Figure 2, we observe the dynamic nature of testing frequency among patients with acute brain injuries (ABI) over the study period, revealing fluctuations and trends that indicate that the overall testing frequency was 72% and consistent amongst the four ABI subgroups.

Table 2 provides the results of the multivariable analysis examining the factors influencing delirium testing among patients with acute brain injuries. Notably, our analysis revealed several key findings, which are highlighted as follows. Age: Compared to patients 18–44 years of age, patients 65–79 years (aOR 0.79 [0.69, 0.90]) and those 80 years and older (aOR 0.58 [0.50, 0.68]) were less likely to undergo delirium testing. Compared to non-White patients, White patients were more likely to be tested for delirium (aOR 1.20 [1.08, 1.34]). Compared to English-speaking patients, non-English-speaking patients (aOR 0.73 [0.64, 0.84]) were less likely to undergo delirium testing. Compared to patients with AIS, patients with SAH (aOR 1.21 [1.04, 1.41]) and TBI (aOR 1.30 [1.16, 1.45]) were more likely to undergo delirium testing. Compared to patients with admission GCS of 13–15, patients with admission GCS of 9–12 (aOR 0.39 [0.34, 0.45]) and those with admission GCS of 3–8 (aOR 0.26 [0.22, 0.30]) were less likely to be tested for delirium. Mechanically ventilated patients (aOR 0.69 [0.60, 0.78]) were less likely to undergo delirium testing. Compared to patients with ICU LOS of 1–7 days, patients with ICU LOS of 8–14 days (aOR 2.57 [2.18, 3.03]) and those with ICU LOS of 15 days or more (aOR 4.61 [3.73, 5.71]) were more likely to undergo delirium testing.

### 3.4. Prevalence and Factors Associated with Delirium

Amongst the 8220 tested, 2217 (27%) tested positive for delirium (AIS: 26.3%, IPH: 32%, SAH: 34.4%, and TBI: 20.4%). For every day in the ICU, the odds of testing positive for delirium were 1.11 [0.10, 0.12]. Table 3 presents the results of the multivariable analysis examining factors associated with diagnosing delirium among patients with ABI. Several significant associations emerged from our analysis. Compared to patients 18–44 years of age, patients 45–64 years (aOR 1.63 [1.38, 1.91]), patients 65–79 years (aOR 2.18 [1.84, 2.59]), and those 80 years and older (aOR 3.18 [2.59, 3.90]) exhibited significant odds of being diagnosed with delirium. Patients with intracranial pressure monitoring (aOR 1.52 [1.28, 1.80]) showed significantly higher odds of being diagnosed with delirium. Compared to patients with the lowest GCS of 13–15, patients with the lowest GCS of 9–12 (aOR 4.90 [4.13, 5.82]) and those with the lowest GCS of 3–8 (aOR 8.31 [6.88, 10.03]) were more likely to be diagnosed with delirium. Compared to patients with ICU LOS of 1–7 days, patients with ICU LOS of 8–14 days (aOR 1.73 [1.46, 2.06]) and those with ICU LOS of 15 days or more (aOR 2.25 [1.84, 2.75]) were more likely to be diagnosed with delirium.

### 3.5. Associations between Delirium, Critical Care Resource Utilization, and Clinical Outcomes

Investigating the impact of delirium on critical care resource utilization, we found significant associations in multiple domains. These are highlighted in Table 4 and summarized here.

### 3.6. Monitoring Utilization

Our univariable and non-adjusted analysis examined the associations between delirium, various monitoring modalities, and medication use. Notably, continuous EEG monitoring was significantly more common among patients diagnosed with delirium (27.0%) compared to those without (9.06%), with an odds ratio (OR) of 3.71 [3.26, 4.22] (*p* < 0.001). Similarly, the utilization of pupillometers was significantly higher in delirium-diagnosed patients (46.2%) compared to non-delirious patients (15.5%), with an OR of 4.69 [4.21, 5.23] (*p* < 0.001). Delirium was also associated with the increased use of medications, such as dexmedetomidine (OR = 5.56), haloperidol (OR = 6.19), quetiapine (OR = 7.47), lorazepam (OR = 2.53), and propranolol (OR = 3.98), all with *p*-values <0.001.

### 3.7. Nonpharmacological Delirium Measures

Patients diagnosed with delirium (23.7%) were more likely to receive nonpharmacological delirium measures compared to non-delirious patients (7.11%), with an OR of 4.05 [3.53, 4.66] (*p* < 0.001).

### 3.8. Complications

Delirium was significantly associated with an increased incidence of complications, including ventilator-associated pneumonia (VAP) (OR = 2.78, *p* < 0.0001), catheter-associated urinary tract infection (CAUTI) (OR = 2.65, *p* < 0.0001), acute respiratory distress syndrome (ARDS) (OR = 1.78, *p* < 0.001), and deep vein thrombosis/pulmonary embolism (DVT/PE) (OR = 1.80, *p* < 0.001).

### 3.9. Discharge GCS

Patients with delirium were discharged with lower Glasgow Coma Scale (GCS) scores (9–12 and 3–8) compared to those with GCS scores of 13–15, and with ORs of 2.98 and 3.97, respectively (both *p* < 0.001).

### 3.10. Discharge Disposition

Delirium had significant implications for discharge disposition. Patients diagnosed with delirium were less likely to be discharged home (28.9%) compared to non-delirious patients (59.1%). Instead, they were more likely to be discharged to rehabilitation facilities (22.0%, OR = 2.75, *p* < 0.001), long-term acute care facilities (6.36%, OR = 2.51, *p* < 0.001), skilled nursing facilities (26.8%, OR = 3.67, *p* < 0.001), or, unfortunately, experienced a higher rate of expiration (15.9%, OR = 7.26, *p* < 0.001), with all associations significant at *p* < 0.001.

## 4. Discussion

Delirium is a complex neuropsychiatric syndrome that often complicates the course of patients with acute brain injuries (ABI). In this single-center retrospective cohort study, we aimed to comprehensively examine the epidemiology, diagnostic testing, associated factors, and clinical implications of delirium in patients with ABI, encompassing acute ischemic stroke (AIS), intraparenchymal hemorrhage (IPH), subarachnoid hemorrhage (SAH), and traumatic brain injury (TBI). Our findings shed light on several critical aspects of delirium in this population, contributing to our understanding of its prevalence, risk factors, and impact on clinical care and outcomes. The main findings of our study are that (1) seven out of ten patients in the neurocritical care unit are tested for delirium, (2) two out of five patients test positive for delirium, (3) delirium is associated with critical care utilization, and (4) delirium is associated with high rates of mortality and lower GCS at discharge from the hospital.

### 4.1. Diagnostic Testing Frequency

Our study finds seven out of ten patients in the neurocritical care unit are assessed for delirium. Patients with advanced age (those 65 years and older and those 80 years and older) are tested less than younger patients. This is contrary to what we would have expected. We expected that the delirium assessment would be higher with advancing age. This finding is significant and identifies a gap in our current practice. The exact frequency of delirium testing may depend upon the individual intensive care unit, but twice-daily assessments (i.e., during nurses’ shifts) would be an accepted best practice. In neurocritically ill patients, reasons for not testing may include a depressed level of consciousness or a low GCS score of 3–12. It is also challenging to assess delirium in those with aphasia and cognitive deficits due to underlying neurological pathology. This is particularly important in neurocritically ill patients since the Confusion Assessment Method for the intensive care unit [20] includes testing for inattention [22], which may be influenced by pathology in the dominant hemisphere. We also identified that non-English language may be a barrier to assessing patients for delirium. In summary, all neurocritically ill patients should be evaluated, and reasons provided why delirium assessments were not performed. 

### 4.2. Delirium Incidence

Our study finds an overall prevalence of delirium to be 27%. When we compared our numbers with those published in the literature, we found that our overall rates are within the pooled rate of 12–43% [19,23,24] and higher than that published by von Hofen-Hohloch and colleagues (18.7%) [25]. Our numbers are higher (26.3% vs. 18.3%), as published by Kozak and colleagues in AIS patients not admitted to an ICU [26]. Our numbers are similar for IPH (32% vs. 27%) to those published by Naidech et al. [27] and lower for TBI (20.4% vs. 60%) compared to those published by Wilson et al. [3]. The prevalence of delirium in our study is lower than those reported in the medical intensive care unit [28]. Our study reaffirmed the significance of delirium as a frequent complication in patients with ABI and specific subtypes of ABI [29]. Specifically, in patients with acute ischemic stroke, dysphagia at the time of admission to an intensive care unit was independently associated with the risk of delirium [30]. Additionally, subtypes of patients with delirium based on the demographics, vital signs, hemodynamic support, laboratory values, ventilation, and sedation may differ in the duration of delirium, shock, renal impairment, coma, and mortality, as demonstrated in a study conducted in a medical or surgical critical care unit [31].

### 4.3. Factors Associated with Delirium

The multivariable analysis highlighted several demographic and clinical factors associated with the diagnosis of delirium in ABI patients. Age, sex, race/ethnicity, preferred language, and pre-admission co-morbidities emerged as significant predictors. These findings underscore the intricate interplay between these factors and their influence on delirium risk. Additionally, our analysis demonstrated that the need for mechanical ventilation, ICP monitoring, and prolonged ICU stays were also linked to an increased likelihood of delirium diagnosis. Understanding these associations can guide targeted interventions and screening protocols to identify high-risk patients and implement preventive measures. What is challenging is to determine the cause or effect of some of these factors on delirium. For example, the length of stay in neurocritically ill patients often depends upon stable neurological examination. Thus, it is conceivable that patients with a higher length of stay in the intensive care unit may be at high risk of delirium. However, delirium and instability in the neurological examination may have increased their length of stay in the intensive care unit. Similarly, the risk of delirium in patients with intracranial pressure monitoring may be high due to many factors (underlying pathology, fluctuating neurological examination, instability due to intracranial pressures), and thus may confound cause and effect. Our study finding that mechanically ventilated patients are at higher risk for delirium is similar to previously published [32,33,34].

### 4.4. Critical Care Utilizations, Their Implications, and Outcomes

Delirium had far-reaching clinical implications in our study population. Patients diagnosed with delirium exhibited heightened critical care resource utilization, including increased use of continuous EEG monitoring and automated pupillometry. Moreover, they were more likely to receive pharmacological interventions to manage pain, agitation, and distress symptoms [35]. This highlights the clinical challenge of managing delirium and underscores the importance of effective delirium prevention and management strategies in ABI patients. Specifically, the medications administered for pain, agitation, and distress and their associations with delirium warrant further discussion. Quetiapine is commonly prescribed in our intensive care unit for agitated delirium; thus, our study’s findings are similar to previously published data [34,36,37,38]. Dexmedetomidine is a commonly prescribed drug to treat agitation in our intensive care unit, especially in patients who are not mechanically ventilated [39]. Our practice seems similar to general critical care unit practices [40] and concurs with the evidence [41]. However, we do not generally restrict its use only at night, where it has been shown to reduce the incidence of delirium [42]. Agitated delirium may have warranted some of these diagnostic and therapeutic interventions, and these interventions by themselves may exacerbate delirium further.

Complications were more prevalent among delirium-diagnosed patients. Sepsis contributes to delirium; thus, the associations between hospital-acquired infections and delirium are not surprising. Often, we send blood and other body fluids to rule out infection in patients with delirium. We emphasize the importance of reducing the risk of infections, which may have a positive outcome in preventing delirium. Lastly, our study revealed significant variations in cognitive status at discharge and discharge disposition based on delirium diagnosis. Patients diagnosed with delirium were less likely to be discharged home and more likely to be transferred to rehabilitation facilities, long-term acute care facilities, skilled nursing facilities, or, regrettably, experienced a higher rate of death. The discharge disposition implies an incomplete recovery, and recovery without minimal disability that can be managed at home by itself or with home health services. These outcomes underscore the comprehensive impact of delirium on neurological recovery and healthcare resource allocation, and every attempt must be made to reduce the incidence of delirium in neurocritically ill patients.

### 4.5. Implications of Findings and Areas for Quality Improvement

The findings of this study have significant implications for quality improvement in the care of ABI patients. There are disparities in delirium testing and diagnosis, particularly in elderly and non-English-speaking patient populations. These disparities highlight opportunities for improvement in healthcare delivery. With advancing age, we found less delirium testing but higher odds of testing positive for delirium, underscoring the importance of prioritizing delirium testing in this high-risk patient population. This recommendation aligns with the American Academy of Neurology’s proposed quality measure set [43]. Similarly, the use of nonpharmacological delirium precautions was also underutilized [43,44]. Nonpharmacological anti-delirium measures may have potential benefits [45] in reducing delirium incidence and preventing falls, with a trend toward decreasing length of stay in the adults hospitalized in the ICU and non-ICU [46]. We identify this as an area for improvement at our hospital. The CAM-ICU is a standard delirium screening tool. Still, it may be at risk for reliability issues in patients receiving sedation [47], which may partly explain the low rates of delirium screening in intubated and mechanically ventilated patients. Strategies such as targeted educational initiatives, language services, and cultural competency training can be implemented to address these disparities. The 4AT scoring has been proposed as a simple scoring test in geriatric patients, especially those with dementia and who are non-English speaking [48]. Education for neurocritical care clinicians (nurses) has yielded positive results in a sustained reduction in the rates of delirium [23]. Additionally, benchmarking with other healthcare institutions and reviewing the published literature can help identify best practices and ensure that care aligns with evidence-based guidelines. Addressing these disparities and continuously monitoring and adapting strategies can enhance the quality of care provided to ABI patients, reduce inequities in testing and diagnosis, and ultimately improve patient outcomes. Finally, we cannot remiss the role hourly neurological assessments may play in adding to the factors associated with delirium for our high-risk ABI patients. While our study did not specifically examine the clinical trajectory of neurological assessment frequency, future work in delirium testing and interventions may allow a more nuanced examination. A recent study demonstrated that patients with TBI with more frequent neurochecks had a higher risk of developing delirium compared to those with less frequent neurochecks [24]. The researchers noted that after accounting for demographics, comorbidities, and injury patterns, patients with hourly neurochecks had the greatest rate of delirium compared to those with two- or four-hourly neurochecks [24]. Thus, while hourly neurological examination may be beneficial in the acute phase of neurological injury, prolonged use may be parodically harmful due to sleep deprivation [49,50].

### 4.6. Study Strengths and Limitations

The strengths of this study are underscored by a substantial sample size, encompassing a diverse cohort of patients with ABI, and a lengthy study period spanning over eight years. Including a large sample of patients enhances our analyses’ statistical power and precision, allowing us to draw more robust conclusions. Additionally, the extended study duration allows us to capture temporal trends and assess potential changes in clinical practices and outcomes over the years. Our study has several limitations, including its retrospective nature and reliance on electronic health records, which were not created solely for clinical research, which may lead to potential selection bias and missing data. Additionally, this study was conducted at a single academic medical center, limiting generalizability to broader patient populations. Future research should explore the effectiveness of targeted interventions and prevention strategies to mitigate delirium risk in ABI patients and improve clinical outcomes.

## 5. Conclusions

In this single-center study, we find that seven out of ten patients in the neurocritical care unit are tested for delirium, and approximately two out of every five patients test positive for delirium. We demonstrate disparities in delirium testing by age and preferred language, identified high-risk subgroups, and the association between delirium, critical care resource use, complications, discharge GCS, and disposition. Prioritizing equitable testing and diagnosis, especially for elderly and non-English-speaking patients, is crucial for delivering quality care to this vulnerable group. In conclusion, delirium is a prevalent and complex issue in patients with ABI, encompassing a range of subtypes. This study contributes valuable insights into the epidemiology, diagnostic testing, associated factors, and clinical implications of delirium in this population. These findings underscore the need for proactive screening, prevention, and management strategies to optimize the care and outcomes of ABI patients while also highlighting avenues for further research in this critically important area of neurocritical care.

## Figures and Tables

**Table 1:** Study cohort characteristics.

	Study Sample
	(N = 11,332)
Age	
18–44 years	2253 (19.9%)
45–64 years	3878 (34.2%)
65–79 years	3424 (30.2%)
80 years and above	1777 (15.7%)
Sex	
Female	4618 (40.8%)
Male	6714 (59.2%)
Race/ethnicity	
Non-White	2618 (23.1%)
White	8714 (76.9%)
Language preference	
English	10,014 (88.4%)
Non-English	1318 (11.6%)
Admitting diagnosis	
Acute ischemic stroke	4146 (36.6%)
Spontaneous intracerebral hemorrhage	2433 (21.5%)
Subarachnoid hemorrhage	1595 (14.1%)
Traumatic brain injury	3158 (27.9%)
Pre-admission comorbidities	
Dementia	639 (5.6%)
Psychoses	650 (5.7%)
Depression	1817 (16.0%)
Alcohol abuse	1359 (12.0%)
Drug abuse	763 (6.7%)
Admission Glasgow Coma Score (GCS) category	
Admission GCS 3–8	3102 (27.4%)
Admission GCS 9–12	1413 (12.5%)
Admission GCS 13–15	6817 (60.2%)
Mechanical ventilation	4704 (41.5%)
Intracranial pressure monitoring	2044 (18.0%)
Intensive care unit length of stay	
1–7 days	9023 (79.6%)
8–14 days	1298 (11.5%)
15 days or more	1011 (8.90%)

**Table 2:** Multivariable analysis of factors influencing delirium testing in patients with acute brain injuries.

	aOR [95% CI]	Bonferroni Corrected *p*-Value
Age groups		
18–44 years	Reference	
45–64 years	0.93 [0.82, 1.06]	0.1989
65–79 years	0.79 [0.69, 0.90]	<0.0001
≥80 years	0.58 [0.50, 0.68]	<0.0001
White race	1.20 [1.08, 1.34]	0.0001
Non-English language preference	0.73 [0.64, 0.84]	0.0000
Admission diagnosis		
Acute ischemic stroke	Reference	
Spontaneous intracerebral hemorrhage	0.96 [0.86, 1.08]	0.5378
Subarachnoid hemorrhage	1.21 [1.04, 1.41]	0.0014
Traumatic brain injury	1.30 [1.16, 1.45]	<0.0001
Admission Glasgow Coma Scale Score	1.18 [1.16, 1.19]	<0.0001
Admission GCS 13–15	Reference	
Admission GCS 9–12	0.39 [0.34, 0.45]	<0.0001
Admission GCS 3–8	0.26 [0.22, 0.30]	<0.0001
Intracranial pressure monitoring	1.03 [0.89, 1.19]	0.4584
Mechanical ventilation	0.69 [0.60, 0.78]	<0.0001
ICU Length of stay		
1–7 days	Reference	
8–14 days	2.57 [2.18, 3.03]	<0.0001
15 days and more	4.61 [3.73, 5.71]	<0.0001

Abbreviations: ICU—intensive care unit. Notes: During univariable analysis, sex and pre-admission diagnosis were not significant and dropped from the multivariable analysis. Hosmer–Lemeshow multivariable model goodness-of-fit test: *p*-value = 0.05.

**Table 3:** Factors associated with the diagnosis of delirium in patients with acute brain injuries (multivariable analysis).

Study Sample n = 8220	aOR [95% CI]	Bonferroni Corrected *p*-Value
Age groups		
18–44 years	Reference	
45–64 years	1.63 [1.38, 1.91]	<0.0001
65–79 years	2.18 [1.84, 2.59]	<0.0001
80 years and above	3.18 [2.59, 3.90]	<0.0001
White race	1.19 [1.04, 1.38]	0.0144
Non-English	1.04 [0.86, 1.25]	0.6925
Pre-admission co-morbidities		
Psychosis	1.01 [0.78, 1.31]	0.9359
Depression	0.85 [0.73, 1.00]	0.0560
Alcohol use	1.19 [0.99, 1.43]	0.0610
Substance use	1.16 [0.92, 1.48]	0.2164
Admitting diagnosis		
Acute ischemic stroke	Reference	
Spontaneous intracerebral hemorrhage	1.63 [1.40, 1.90]	<0.0001
Subarachnoid hemorrhage	1.15 [0.96, 1.36]	0.1242
Traumatic brain injury	1.16 [1.00, 1.35]	0.0529
Lowest GCS category		
Lowest GCS 13–15	Reference	
Lowest GCS 9–12	4.90 [4.13, 5.82]	<0.0001
Lowest GCS 3–8	8.31 [6.88, 10.03]	<0.0001
Intracranial pressure monitoring	1.52 [1.28, 1.80]	<0.0001
Mechanical ventilation	1.09 [0.93, 1.28]	0.2740
ICU length of stay		
1–7 days	Reference	
8–14 days	1.73 [1.46, 2.06]	<0.0001
15 days and more	2.25 [1.84, 2.75]	<0.0001

Notes: Lowest GCS category—defined as the lowest Glasgow Coma Score recorded during the intensive care unit stay. During the univariable analysis, sex was not significant, so it was dropped from the multivariable analysis. Hosmer–Lemeshow multivariable model goodness-of-fit test: *p*-value = 0.3744.

**Table 4:** Univariable analysis of associations between delirium, critical care resource utilization, and clinical outcomes.

	No Delirium	Delirium	Odds Ratio [95% CI]	Bonferroni Corrected *p*-Value
	N = 6003	N = 2217		
Monitoring utilization				
Brief EEG	686 (11.4%)	524 (23.6%)	2.40 [2.11, 2.72]	<0.001
Continuous EEG	544 (9.06%)	598 (27.0%)	3.71 [3.26, 4.22]	<0.001
Pupillometer	928 (15.5%)	1024 (46.2%)	4.69 [4.21, 5.23]	<0.001
Medication use				
Dexmedetomidine	234 (3.90%)	408 (18.4%)	5.56 [4.70, 6.59]	<0.001
Haloperidol	285 (4.75%)	523 (23.6%)	6.19 [5.31, 7.23]	<0.001
Quetiapine	400 (6.66%)	771 (34.8%)	7.47 [6.53, 8.54]	<0.001
Lorazepam	1240 (20.7%)	881 (39.7%)	2.53 [2.28, 2.81]	<0.001
Propranolol	194 (3.23%)	260 (11.7%)	3.98 [3.28, 4.83]	<0.001
Oral morphine equivalent use	238 (1058)	1082 (18612)	1.00 [1.00, 1.00]	0.053
Nonpharmacological delirium measures	427 (7.11%)	525 (23.7%)	4.05 [3.53, 4.66]	<0.001
Complications				
VAP	116 (1.93%)	115 (5.19%)	2.78 [2.13, 3.61]	<0.0001
CAUTI	75 (1.25%)	72 (3.25%)	2.65 [1.91, 3.68]	<0.0001
ARDS	0.07 (0.25)	0.12 (0.32)	1.78 [1.51, 2.10]	<0.001
DVT/PE	207 (3.45%)	134 (6.04%)	1.80 [1.44, 2.25]	<0.001
Discharge GCS				
13–15	5599 (93.3%)	1769 (79.8%)	Ref.	Ref.
9–12	187 (3.12%)	176 (7.94%)	2.98 [2.41, 3.68]	<0.0001
3–8	217 (3.61%)	272 (12.3%)	3.97 [3.29, 4.78]	<0.0001
Discharge disposition				
Home	3547 (59.1%)	641 (28.9%)	Ref.	Ref.
Rehabilitation facility	981 (16.3%)	488 (22.0%)	2.75 [2.40, 3.16]	<0.0001
Acute care facility	311 (5.18%)	141 (6.36%)	2.51 [2.02, 3.11]	<0.001
Skilled nursing facility	896 (14.9%)	595 (26.8%)	3.67 [3.21, 4.20]	<0.0001
Expired	268 (4.46%)	352 (15.9%)	7.26 [6.07, 8.70]	<0.0001

Abbreviations: EEG—electroencephalography; VAP—ventilator-associated pneumonia; CAUTI—catheter-associated urinary tract infection; ARDS—adult respiratory distress syndrome; DVT/PE—deep venous thrombosis/pulmonary embolism.

## Data Availability

Data used for this study are not publicly available.
